# Stimulating Parenting Practices in Indigenous and Non-Indigenous Mexican Communities

**DOI:** 10.3390/ijerph15010029

**Published:** 2017-12-25

**Authors:** Heather A. Knauer, Emily J. Ozer, William Dow, Lia C. H. Fernald

**Affiliations:** School of Public Health, University of California, Berkeley, CA 94720, USA; eozer@berkeley.edu (E.J.O.); wdow@berkeley.edu (W.D.); fernald@berkeley.edu (L.C.H.F.)

**Keywords:** parenting, indigenous, poverty, early childhood development

## Abstract

Parenting may be influenced by ethnicity; marginalization; education; and poverty. A critical but unexamined question is how these factors may interact to compromise or support parenting practices in ethnic minority communities. This analysis examined associations between mothers’ stimulating parenting practices and a range of child-level (age; sex; and cognitive and socio-emotional development); household-level (indigenous ethnicity; poverty; and parental education); and community-level (economic marginalization and majority indigenous population) variables among 1893 children ages 4–18 months in poor; rural communities in Mexico. We also explored modifiers of associations between living in an indigenous community and parenting. Key findings were that stimulating parenting was negatively associated with living in an indigenous community or family self-identification as indigenous (β = −4.25; SE (Standard Error) = 0.98; β = −1.58; SE = 0.83 respectively). However; living in an indigenous community was associated with significantly more stimulating parenting among indigenous families than living in a non-indigenous community (β = 2.96; SE = 1.25). Maternal education was positively associated with stimulating parenting only in indigenous communities; and household crowding was negatively associated with stimulating parenting only in non-indigenous communities. Mothers’ parenting practices were not associated with child sex; father’s residential status; education; or community marginalization. Our findings demonstrate that despite greater community marginalization; living in an indigenous community is protective for stimulating parenting practices of indigenous mothers.

## 1. Introduction

Stimulating parenting practices and enriching home environments can promote and sustain positive child development [1]. In low- and middle-income countries (LMICs), these stimulating parental and environmental factors can be particularly important for supporting children and buffering them from negative exposures that could hurt cognitive and language development. The interplay among ethnicity, poverty, parenting, and child development in ethnic minority populations in LMICs is not well understood and has significant implications for programs or policies aimed at improving outcomes for children living in poverty. To address this gap, this study will examine differences in stimulating parenting behaviors between indigenous and non-indigenous communities. Within each community type, we will examine: (a) the demographic characteristics of mothers who engage in more supportive and stimulating behaviors and who provide more enriching home learning environments for their children; and (b) the association between these supportive behaviors and ethnic identity in poor, rural indigenous and non-indigenous communities in Mexico.

### 1.1. Cultural Influences on Parenting and Child Development

Parenting practices that have been most consistently associated with optimal child cognitive and socioemotional development, described here as stimulating parenting, include reading books, telling stories, engaging in active play, and avoiding harsh punishment [2,3,4]. In European American contexts, authoritative parenting promotes optimal child socio-emotional development or school performance [5,6,7,8]. However, cross-cultural research in child development across socioeconomic, racial, and ethnic contexts demonstrates that authoritative parenting is not consistently associated with better child cognitive or social development, or academic achievement [9,10,11,12,13]. Cultural values and beliefs influence parental goals and attitudes toward their child’s behavior and development [14]. As a result, differing cultural and ethnic values and beliefs may lead to different parenting behaviors to promote child development [15,16]. Therefore, it is important to examine the cultural, socioeconomic and ethnic contexts in which parenting and child development are occurring [17,18,19].

In LMICs, the literature on the association between parenting practices and child cognitive, language, and motor development are not conclusive. Two large studies in Bangladesh and Ecuador have demonstrated a positive association between parents who display more stimulating behaviors and child development, using less in-depth measures of parenting (family care indicators, and a subset of the Home Observation for Measurement of the Environment (HOME), respectively [20,21], while studies in Paraguay and Costa Rica, with much smaller sample sizes but using the full HOME Inventory, did not [22,23]. In addition to these concerns, existing studies in LMICs have not yet distinguished among ethnic or indigenous groups to examine if the relation between parenting practices and child development may be different among minority ethnic groups. 

### 1.2. Parenting Styles and Practices in Mexico

Ethnographic research has identified differences in parenting styles in Mexico between a more conservative and authoritarian “traditional” style more common to rural areas, and a more open and authoritative “counter-culture” style common to urban areas [24,25]. Several studies have examined whether urban Mexican parents’ beliefs have transitioned away from traditional parenting styles for mothers and fathers [26,27,28]. The evolving evidence on parenting in Mexico highlights that in addition to socio-economic status (SES) and education, ethnicity and rurality may also influence parenting practices in Mexico [24]. However, previous studies have largely treated parents in Mexico as a single ethnic group and have taken place in urban areas [29,30], without explicit examination of Mexico’s demographic diversity in ethnicity and rurality.

### 1.3. Diverse Populations of Mexico

Mexico has the largest Spanish-speaking population and the largest and most diverse indigenous population in Latin America [24,31]. There are 62 recognized indigenous languages spoken by nearly 13 million people, with 15% of the Mexican population over the age of three self-identified as indigenous [32]. Nearly 80% of indigenous people live in the southern region of Mexico and primarily live in rural communities with less than 15,000 inhabitants [31]. 

Studying indigenous populations is important because indigenous populations are often neglected in health research, yet have poorer social and health outcomes than non-indigenous populations [33] that are rooted in poverty and social inequities [34,35]. One example of a large social inequity is that the prevalence of adult illiteracy is nearly three times greater among indigenous people in Mexico compared to the national rate (6.3%) [36]. Indigenous populations have less access to health care and are in poorer health than non-indigenous populations [37], with notably higher rates of infant mortality, malnutrition, and stunting among children [31,33,36]. 

Much of the literature on indigenous populations focuses on poverty and marginalization, but it is unclear how these factors may affect parenting practices, or whether there are aspects of parenting in indigenous communities that may be protective. It is also unclear how indigenous ethnicity may affect parenting practices, and how these practices in turn relate to child development. Despite the large indigenous population in Mexico, studies on parenting have not examined contemporary differences between indigenous and non-indigenous populations, focusing instead on temporal cultural evolution or comparisons with Mexican American and European American populations [29]. There is some evidence that parenting practices may differ across indigenous and non-indigenous families. For example, in a small study of young indigenous Mayan children in Guatemala, parents gave children younger than five more freedom to act in their self-interest rather than aligning with the larger collaborative culture. Parents attributed children’s behaviors to their cognitive and socio-emotional immaturity, which they believed would later mature to conform to cooperative cultural beliefs, instead of expecting children’s behaviors to conform to cultural expectations from an early age, as is common in the United States [38]. 

### 1.4. Current Study

The present study has three main objectives. For our first objective, we tested differences in Mothers’ stimulating parenting behaviors between indigenous and non-indigenous communities. We hypothesize that there will be a negative association between stimulating parenting practices and indigenous status beyond that accounted for by community economic marginalization and individual SES. The second objective is to explore demographic characteristics associated with mothers’ parenting practices within indigenous and non-indigenous communities, and how demographic and SES factors may modify the relation between living in an indigenous community and parenting practices. To address our second objective, we stratified the analyses by indigenous and non-indigenous communities to examine the associations between child, family and community characteristics and parenting practices. Community stratification was part of the original study design to examine differential effects of a parenting intervention between indigenous and non-indigenous communities, which allowed us to conduct this analysis. Because indigenous communities are more marginalized than non-indigenous communities and have less access to services and resources (such as children’s books in indigenous languages), we hypothesize that SES may be less strongly associated with parenting practices in indigenous communities than in non-indigenous communities where greater SES may mean greater access to resources within the community. For our third objective, we examined modifiers of the association between parenting practices and living in an indigenous community in separate models. We hypothesize there to be a differential association owing to relatively greater marginalization or stigma associated with being an ethnic minority family living in an ethnic majority community as compared to an ethnic minority community.

## 2. Materials and Methods

### 2.1. Study Design

This secondary data analysis uses baseline data from a randomized controlled effectiveness evaluation of a parenting education program, Educación Inicial, operating in collaboration with a national conditional cash transfer (CCT) program (Prospera) in rural communities in three states (Puebla, Oaxaca, and Chiapas) in Mexico. Details of the results from the Randomized controlled trial (RCT) and parenting program can be found in the impact evaluation, [39] and mediation analysis [40]. This study was approved by the institutional review boards at the University of California, Berkeley and the National Institute of Public Health in Cuernavaca, Mexico (protocol ID: 2010-05-1528). 

### 2.2. Sampling and Data Collection

A sample of 102 indigenous and 102 non-indigenous communities were selected for randomization according to the following study eligibility criteria: (1) rural location (population < 2500) in the Mexican states of Chiapas, Puebla, or Oaxaca; (2) at least 15 families with children ages 0–2 years; (3) at least 70% of families in the community eligible to receive CCT benefits; and (4) no current or prior operation (within the past five years) of Educación Inicial. 

All CCT program beneficiary families in the eligible indigenous and non-indigenous communities were invited to participate in the study. Baseline data were collected on 2472 children ages 4–18 months in 2008, and 1929 children (78%) were eligible for a developmental assessment. Of the eligible children, one was lost due to missing the HOME Inventory, five were lost due to missing the Extended Ages and Stages Questionnaire, and 34 were missing the Ages and Stages Socio-Emotional Questionnaire. In several cases, children were missing more than one measure, which resulted in an analysis sample of 1893 children. We retained the original RCT evaluation sample for this secondary data analysis. 

### 2.3. Measurement of Parenting Practices

Stimulating parenting practices were measured using the Infant/Toddler version of The Home Observation for Measurement of the Environment (HOME) Inventory [41]. The Infant/Toddler version of the HOME is applied to children ages 0–3 years, is composed of 45 items scored as yes or no, and is conducted through an in-home observation and interview of the parent (in this study, the mother was interviewed). A higher score on the HOME indicates more supportive and stimulating parenting practices, although the instrument does not set any screening cutoff points. The HOME has six subscales: (1) parent responsiveness to child’s behavior (Responsivity subscale); (2) parent avoidance of inappropriate restriction and punishment (Acceptance subscale); (3) family routines and safety and predictability of the home’s physical environment (Organization subscale); (4) play and learning materials in the home for development (Learning Materials subscale); (5) parental active engagement in the child’s development (Involvement subscale); and (6) parental involvement of a variety of people and experiences in the child’s daily life (Variety subscale) [42,43]. The items that comprise the instrument were selected based on empirical evidence, and then validated through testing [43]. The instrument has been well validated in the U.S. and used worldwide, including in several Latin American countries [44,45]. We conducted an exploratory factor analysis that supported the use of the six subscales in our population; the individual items loaded onto six factors that cumulatively explained the variance in HOME subscale scores. Conservative imputation of zero was applied to retain a score for the total HOME for children missing 1–3 questions (74 missing 1 item, 8 missing 2 items, and 6 missing 3 items). We chose a more conservative imputation since this is our primary outcome of interest. Less than 5% of the analysis sample (*n* = 88) was missing between 1 and 3 questions on the HOME; only one child was missing more than three questions on the HOME, and was therefore excluded from analysis. The internal consistency of the scale was satisfactory (Cronbach α = 0.8227).

### 2.4. Measurement of Child Development

Child’s baseline cognitive development was assessed using the Extended Ages and Stages Questionnaire (EASQ), a developmental screening tool used globally. The EASQ was adapted for use in Mexico by researchers at the Instituto Nacional de Perinatología in Mexico City, and administered to parents as an interview. The EASQ is designed for children 4–60 months old and measures three developmental domains: gross motor function, personal–social ability, and communication; individual domain scores are summed to produce a global score [46]. The Ages and Stages Questionnaire: Socio-Emotional (ASQ:SE) is a screener for children at risk of emotional or social problems [47]. Missing scores were imputed using the mean of the child’s domain score for any child missing fewer than 3 questions in a given domain. EASQ and ASQ:SE scores were standardized using two-month age intervals with a mean of 100 and standard deviation of 15. 

### 2.5. Measurement of Household, Parent, and Child Characteristics

Data were collected on child age (in months) and sex (boy, girl), parent education (kindergarten or less, completed primary school, completed secondary school or higher), whether the father was present in the household, family self-identification as indigenous, household crowding (number of people in the house divided by the number of rooms), state of residence (Chiapas, Oaxaca, or Puebla), and household wealth. Household wealth was measured using a household asset index, that was consolidated using principal components analysis [39,40], and then divided into quartiles, to identify differences between income groups. Fewer than 1% of follow-up observations were missing data for mother’s education, while 4.8% of observations were missing data for father’s education, and 5.2% for father’s presence. No data were missing for child age or sex, household wealth or crowding, family self-identification as indigenous, or state of residence. Where demographic data were missing, values were imputed using the community mean. In addition to the above measures, household composition (number of adults and children in the house) and the availability of electricity and piped water in the home were used to describe baseline differences between indigenous and non-indigenous communities, but were not included in the analyses.

### 2.6. Measurement of Community Characteristics

A community was defined as indigenous or non-indigenous based on the prominence of the indigenous population. Indigenous communities were classified as such, by National Institute of Statistics and Geography (INEGI), if at least 70% of the population speaks an indigenous language. The community marginalization index, developed by the National Population Council, was used as a measure of community level SES to account for differing SES levels among indigenous and non-indigenous communities. It is a composite measure of 8 indicators (e.g., percent of the population younger than 15 years old that is illiterate or has not completed primary school; average number of occupants per room in households; percent of households with dirt floors; and percent of households without: a toilet, electricity, piped water) that are consolidated using a principal components analysis [48].

### 2.7. Statistical Analysis

For our first objective, we tested differences in the HOME subscale and total HOME scores between indigenous and non-indigenous communities using three techniques determined by type of outcome variable: ordinary least squares regression for the total HOME score; Poisson regressions for the Acceptance, Organization, Involvement, and Variety scales; and negative binomial regressions for the Responsivity and Learning scales. We examined unadjusted means and then adjusted for child age and sex, parent education, father residence in the household, family self-identification as indigenous, household crowding and wealth, and community marginalization. We used Bonferroni corrections to account for multiple tests (Per Comparison Error Rate: 0.05/7 = *p* < 0.007). 

To address our second objective, we stratified analyses of predictive characteristics of total HOME score by community indigenous status. We first examined the unadjusted associations between each of the characteristics and total HOME score. We then analyzed an adjusted model that included demographic characteristics (family self-identification as indigenous, child age and sex, parent education, father’s presence, household wealth and crowding, and community marginalization). 

For our third objective, we examined modifiers of the association between parenting practices and living in an indigenous community, including self-identification as indigenous, parent education, and household wealth. We examined these factors in separate models to further understand how they may moderate the relation between living in an indigenous community and total HOME score. The modification models were adjusted for demographic characteristics. In all of our analyses, we included indicator variables for state of residence (Chiapas, Oaxaca, and Puebla) and clustered standard errors at the community level. We conducted our statistical analyses using STATA 14 (STATA Corporation, College Station, TX, USA).

## 3. Results

### 3.1. Descriptive Statistics

Children in indigenous communities had significantly lower developmental scores and were younger than children in non-indigenous communities (Table 1). Mothers in indigenous communities reported lower completed educational levels than mothers in non-indigenous communities, but fathers in indigenous and non-indigenous communities reported similar educational levels. Fathers were more likely to be present in households in indigenous communities than non-indigenous. Households in indigenous communities reported substantially lower levels of wealth and a greater number of household members with more crowding. While both indigenous and non-indigenous communities were rural, had small populations (<2500), and had high percentages of CCT program beneficiaries (>70%), indigenous communities were significantly more economically marginalized than non-indigenous communities. However, there was no significant difference in the availability of piped water or electricity between indigenous and non-indigenous communities. 

Community indigenous status was based on the proportion (≥70%) of indigenous-language-speaking households in the community. Thus, not surprisingly, 99.81% (*n* = 1051) of households in indigenous communities spoke an indigenous language, and 0.19% (*n* = 2) did not, while in non-indigenous communities, 11.79% (*n* = 99) did and 88.21% (*n* = 741) did not. When indigenousness was measured by self-identification rather than language, a greater number of households in non-indigenous as well as indigenous communities identified as non-indigenous (9.64% or *n* = 81 in non-indigenous communities, and 3.52% or *n* = 37 in indigenous communities; Table 1). Self-identification may be biased by social desirability to be considered part of the majority population, but may also reflect demographic trends of intermarriage or that non-indigenous households may speak an indigenous language if they live in a majority indigenous community, as indigenous communities can have a large non-indigenous population (up to 30%). 

### 3.2. Differences in Stimulating Parenting Practices between Indigenous and Non-Indigenous Communities

Table 2 gives the overall distribution of parenting scores in the population. The mean HOME score for the sample was 25.32, with a standard deviation of 6.08. Table 3 shows mean differences in parenting practices between indigenous and non-indigenous communities, including unadjusted and adjusted total HOME scores and subscale scores. Scores were adjusted for child age and sex, parent education, father’s presence, household wealth and crowding, family self-identification as indigenous, and community marginalization. The unadjusted Responsivity, Organization, Learning, Involvement, and total scores were significantly different between indigenous and non-indigenous communities, even after accounting for Bonferroni correction for multiple testing, but the Acceptance subscale was not. The differences between indigenous and non-indigenous communities for the Acceptance subscale became significant after adjusting for demographic characteristics, but only the Organization, Learning, and Involvement subscales and total HOME score remained significant after demographic adjustment and taking multiple outcomes corrections into account. 

### 3.3. Characteristics Associated with Variability in Stimulating Parenting Practices

Stratified analyses of indigenous and non-indigenous communities (Table 4 and Table 5) show how child, family, and community characteristics were associated with variability in summary parenting scores, and demonstrate the different associations between these factors and parenting among communities. Families in indigenous communities with older children, higher levels of parental educational attainment, greater household wealth, and less household crowding reported higher total HOME scores in unadjusted analyses (Table 4). Adjusted for demographic characteristics, the relation between stimulating parenting practices and families with older children, greater maternal educational attainment, and more household wealth remained significant. Families with mothers who had completed primary school reported total HOME scores nearly 1.5 points higher (β = 1.46, SE = 0.36, *p* < 0.001) than families in which mothers’ education level was kindergarten or less. Total HOME scores were almost three points higher (β = 2.73, SE = 0.68, *p* < 0.001) in families with mothers who completed secondary school or higher compared with those in which mothers had completed kindergarten or less. Households with wealth scores above the 25th percentile reported significantly more stimulating parenting practices than households in the bottom quartile. After adjustment, father’s level of educational attainment and amount of household crowding were not associated with stimulating parenting practices in families living in indigenous communities. Stimulating parenting practices did not differ between boys and girls, whether the father was present in the household, or by degree of community marginalization in either unadjusted or adjusted regressions. 

In comparison, families in non-indigenous communities with older children, mothers who had completed at least secondary school, and reported household wealth above the median also reported significantly more stimulating parenting practices in unadjusted models (Table 5). Families in which the head of household self-identified as indigenous, that had greater household crowding, and that lived in communities that were more marginalized reported less stimulating parenting practices. Adjusted for demographic characteristics, the relation between families with older children, household wealth above the median, and less crowding, and higher reported total HOME score remained significant. Child age was similarly associated with total HOME scores in both indigenous (I) communities as non-indigenous (NI) communities (I: β = 0.28, SE = 0.04, and NI: β = 0.21, SE = 0.04, *p* < 0.001 for both). Household wealth was more strongly associated with total HOME score in non-indigenous communities among the top two quartiles (3rd: β = 3.04, SE = 0.96, *p* = 0.002, 4th: β = 4.14, SE = 0.93, *p* < 0.001) than in indigenous communities (3rd: β = 1.99, SE = 0.48, *p* < 0.001, 4th: β = 1.64, SE = 0.65, *p* = 0.014), but total HOME scores in the second quartile were not consistently significantly higher than those in the bottom quartile. Greater household crowding was inversely associated with stimulating parenting (β = −0.38, SE = 0.12, *p* = 0.002) in families living in non-indigenous communities, but not among those living in indigenous communities. Father’s level of educational attainment was inconsistently associated with parenting practices in families in non-indigenous communities. A Wald F-test of joint significance indicated that father’s education was significantly associated with less stimulating parenting practices in non-indigenous communities (*p* = 0.041), but the association was only significant for fathers’ primary school completion. Mother’s educational attainment, family indigenous self-identification, and community marginalization were not significant after adjustment. Stimulating parenting practices did not differ between boys and girls, or whether the father was present in the household in any of the analyses among non-indigenous communities. 

### 3.4. Effect Modifiers of the Association between Indigenous Community and Total HOME Score

Table 6 displays modifiers of the association between indigenous community and parenting, including indigenous self-identification, parent education, and household wealth. All modification models were adjusted for demographic characteristics. In the stratified analyses, families that identified as indigenous reported significantly lower total HOME scores in non-indigenous communities (Table 5). In contrast, in indigenous communities, families that identified as indigenous reported marginally higher total HOME scores (Table 4). When the interaction between living in an indigenous community and family self-identification as indigenous was examined, indigenous community and indigenous self-identification were both independently associated with lower total HOME score (β = −4.25, SE = 0.98, *p* < 0.001; β = −1.58, SE = 0.83, *p* = 0.060, respectively); however, families that identified as indigenous and lived in indigenous communities had statistically significantly higher HOME scores as compared to indigenous families that lived in non- indigenous communities (β = 2.96, SE = 1.25, *p* = 0.019; Table 6). 

The interaction between parent’s educational attainment and living in an indigenous community was significant for both mother’s and father’s primary school completion (β = 1.54, SE = 0.065, *p* = 0.018; β= 1.99, SE= 0.72, *p* = 0.006, respectively) in indigenous communities compared to those who had not completed primary school, but not for completion of secondary school or higher. However, father’s completion of primary school was negatively associated with total HOME score (β = −1.65, SE = 0.61, *p* = 0.007). Household wealth scoring at or above the median was also independently associated with higher HOME scores, but only the interaction between the highest quartile (greater than 75th percentile) had a significant interaction with indigenous community, and the direction of the effect was negative (β = −3.14, SE = 1.17, *p* = 0.008). When the wealth–indigenous interaction was included in the model, living in an indigenous community was no longer independently associated with total HOME score (Table 6). 

## 4. Discussion

To our knowledge, this is the first large-scale study to examine the relationship between parenting (as measured rigorously by both interview and observations) and indigenousness in a diverse sample of indigenous and non-indigenous families living in a range of communities with varying proportions of indigenous families. The key study findings were that, while living in an indigenous community and family self-identification were independently associated with less stimulating parenting practices, for indigenous families, living in an indigenous community where they were a part of the majority population was associated with more stimulating parenting behaviors compared to living in a non-indigenous community. 

Our first objective was to test differences in Mothers’ stimulating parenting behaviors between indigenous and non-indigenous communities. Our findings supported our hypothesis; the negative association between living in an indigenous community and less stimulating parenting was robust to the inclusion of controls for community marginalization, household poverty, and other demographic characteristics. HOME sub-scale scores for all but the Acceptance scale were lower in majority indigenous communities than in non-indigenous communities. The Acceptance subscale of the HOME is a set of questions around parents’ tolerance of non-optimal behaviors from their child, and their avoidance of harsh punishment or excessive restriction of their child [41]. An example is parents’ avoidance of expressing overt annoyance with or hostility toward the child. Greater parental acceptance in indigenous communities may reflect the findings of Mosier and Rogoff (2003), in how Mayan parents perceive and tolerate the behaviors of their children under the age of six years. That scores differed between indigenous and non-indigenous communities, after controlling for sociodemographic characteristics, indicates that there were factors beyond parental education and poverty that were differentially associated with parenting, in which ethnicity may play a role. Exemplifying this finding, family self-identification as indigenous modified the association between indigenous community and stimulating parenting. 

The second objective was to explore demographic characteristics associated with mothers’ parenting practices within indigenous and non-indigenous communities, and how demographic and SES factors may modify the relation between living in an indigenous community and parenting practices. We were able to conduct this analysis based on the fact that “indigenous community” was defined according to the proportion of the population that spoke or understood an indigenous language, and all eligible communities were stratified by indigenous status prior to study assignment. Demographic characteristics associated with stimulating parenting were similar in indigenous communities to those observed in other studies in LMICs, such as household wealth and maternal education [44]. Wealth was a strong predictor of stimulating parenting practices, particularly in households with wealth greater than the median, but was more strongly associated with stimulating parenting practices in non-indigenous communities among households above the 75th percentile. These findings support the second study hypothesis, that parenting practices in non-indigenous communities were more sensitive to household SES, if defined as household wealth, than in indigenous communities. The difference in the relation between household wealth and stimulating parenting practices may lie in the fact that indigenous households had much lower wealth on average (mean = 0.59 vs. 0.96 index scores) than non-indigenous households, as well as the fact that the economic difference between wealth quartiles may be greater in non-indigenous communities than indigenous communities. This is to say that money matters less in indigenous communities than non-indigenous communities. Potential explanations could lie in a lower income inequality in indigenous communities, or that majority indigenous communities may have a different type of social structure or hierarchy than non-indigenous communities. However, if household SES is defined as parent education, the association is less consistent. For example, maternal education was not significantly associated with stimulating parenting in non-indigenous communities. The findings in the non-indigenous communities were consistent with a gradient effect of wealth on parenting. The findings in indigenous communities did not display a clear gradient, but nevertheless supported the findings of previous literature linking stimulating parenting practices to SES in Mexico [30] and added nuance of the relative importance of family wealth between indigenous and non-indigenous communities. In contrast, community marginalization (controlling for community indigenous status) was not associated with less stimulating parenting practices, suggesting that community-level poverty was not a good indicator of individual-level outcomes, and variations in parenting practices were determined to a greater extent by individual and household characteristics. 

Our final objective was to examine modifiers of the association between parenting practices and living in an indigenous community. We found evidence for differential patterns of parenting among indigenous families depending on where they lived, supporting our hypothesis. Indigenous parents living in non-indigenous communities used less stimulating parenting practices (based on home observation and interview), but indigenous parents living in majority-indigenous communities demonstrated more stimulating parenting practices. There are two possible directions of this association. The first, is that there may be an effect of community on parenting, the second, is that there may be something unique about indigenous families that move out of indigenous communities. Indigenous families that are minorities in their communities may face additional stressors and challenges that can undermine their parenting. It is also possible that there may be greater economic opportunity outside of indigenous communities, leading parenting responsibilities to fall on other providers or household members. Conversely, indigenous families with less stimulating parenting may be selecting to move out of indigenous communities. Less stimulating parenting among indigenous families that live in non-indigenous communities could be a reflection of less social capital. Future studies may examine factors that help explain the direction and causes of this pattern. 

The extensive use of the HOME Inventory—an in-depth measure used here—across several decades enables us to compare scores for our sample with those from other contexts. The mean total HOME score for this population (mean = 25.3, SD = 6.1) was lower than the original HOME sample from Arkansas in 1979 (60% African American, 40% European American) with a mean of 30.9 and SD of 7.6 [49], as well as a mixed sample from multiple U.S. sites (54% European American, 18% African American, 28% Mexican-American) with a mean of 32.7 and SD of 7.1 [45]. Prior studies in Latin American countries have generally supported use of the HOME Inventory [23,50,51,52], and scores for this sample were similar to those found in other Central and South American countries, with similar income levels at their respective times of study [53]. The mean total score of this study was similar to that of some lower income countries, such as Paraguay [23], and some upper middle-income countries, such as Argentina, Brazil and Uruguay [44,50,54]. However, the mean score for this Mexican sample was lower than a sample in Costa Rica, a lower-income country (at the time of study) [22], and a sample in Chile, another upper middle-income country [55]. HOME scores may be similar across Latin American countries with similar collectivist values. Differences may be due to the particular population studied within Mexico: rural, poor, and marginalized indigenous and non-indigenous communities. 

Strengths of the present study included having a very large sample of both indigenous and non-indigenous families living in a range of majority-indigenous and non-indigenous communities—with excellent assessment of family environment and resources. This design enabled us to examine the parenting patterns of indigenous- and non-indigenous-identifying families living in majority or minority indigenous communities. There are several additional potential design and methodological limitations to this study. Notably, as this is a cross-sectional study, we cannot make claims regarding the directionality of some of the associations found. In addition, the HOME Inventory—while extensive and considered the highest assessment standard for the field—does not capture every aspect of parenting that could potentially influence child development [44]. The HOME was designed to capture key elements of parenting and the home learning environment that are associated with child development; through its use around the world, the HOME has identified similarities and differences in parenting practices between cultures and ethnicities [56]. However, as detailed previously, these associations with child development have not been examined among indigenous populations. There is also a potential for measurement bias of the HOME in indigenous communities, or the HOME may favor a majority culture or ethnicity. However, there was considerable variability in scores, and indigenous families in indigenous communities had higher HOME scores compared to indigenous families in non-indigenous communities. Presumably, indigenous families living in indigenous communities would be less acculturated to the majority culture or ethnicity, countering the notion of measurement bias of the instrument. Nevertheless, there may be selection factors present in the study that are not accounted for. Indigenous families that remain in indigenous communities rather than migrating might be those with stronger economic and social supports, while those families that migrated may be those more likely to have less stimulating parenting practices for reasons not captured in this study. Indigenous families living in non-indigenous communities may also face stigma or other social marginalization not able to be captured in this study. 

Despite these limitations, this study makes a unique contribution to the literature on indigenous marginalization, demonstrating that there are concerns in indigenous communities beyond material wealth and education. Families living in indigenous communities on average had lower parenting scores than families living in non-indigenous communities, suggesting that indigenous communities could be targeted for parenting intervention. However, indigenous families living in indigenous communities had more stimulating parenting practices than indigenous families living in non-indigenous communities. These associations speak to complex relations between ethnicity and place that warrant further investigation. 

## 5. Conclusions

The large-scale of this study and use of an in-depth parenting measure, is uncommon among parenting studies in LMIC (typically only small studies employ the HOME, while larger studies use short, self-reported measures), and the first of its kind among indigenous populations. To our knowledge, this is the first comparative study to examine stimulating parenting practices among indigenous families living in a range of majority and minority indigenous communities in a LMIC. There are few other studies of parenting in indigenous communities, and the existent research has not focused on stimulating parenting behaviors. For example, studies in China have focused on parenting style (warmth and control) [57], and those in the US and Australia have focused on the effects of parenting interventions on child outcomes [58]. We have not found any similar studies in low- and middle- income countries. Children in LMIC are at risk of poor developmental outcomes as a result of poverty [59]. These risks are arguably even greater for children living in marginalized indigenous populations [35]. Indigenous populations warrant being studied, because not studying them contributes to their marginalization. In addition, studying stimulating parenting in minority populations, who are often the focus of early childhood interventions, is important for targeting and curriculum development of social programs aiming to improve parenting practices and the home learning environment. This research agenda is also important for understanding program effects on parenting and how parenting may mediate program effects on early childhood development. This study highlights the parenting practices of indigenous and non-indigenous families living in indigenous or non-indigenous communities, and highlights the need for programming to identify and address the varying difficulties that families may face.

## Figures and Tables

**Table 1:** Characteristics of sample (*n* = 1893).

Characteristic	Non-Indigenous ^1^ *n* = 840		Indigenous ^1^ *n* = 1053		*p*-Value
	Mean	SD or Percent	Mean	SD or Percent	
**Child**					
EASQ scores ^2^					
Communication	102.63	13.90	98.02	15.43	<0.001
Motor	101.65	14.73	98.75	15.10	0.001
Perceptual	102.63	13.55	98.06	15.56	<0.001
Global Score	102.97	14.13	97.79	15.11	<0.001
ASQ Socio-emotional ^2^	102.53	14.48	98.01	14.94	<0.001
Child sex (girl)		49%		49%	0.928
Child age					0.001
4 to 8 months		32%		39%	
9 to 13 months		39%		37%	
14 to 18 months		30%		24%	
**Parent**					
Mother education ^3^					<0.001
Kindergarten or less		13%		27%	
Primary		71%		65%	
Secondary and above		16%		7%	
Father education ^3^					0.105
Kindergarten or less		12%		16%	
Primary		69%		68%	
Secondary and above		19%		16%	
Father resides in home		85%		92%	<0.001
**Household**					
Speaks indigenous language		12%		>99%	<0.001
Indigenous self-identification		10%		96%	<0.001
Piped water		72%		67%	0.472
Electricity		95%		93%	0.585
Household size	6.45	2.31	6.9	2.23	0.008
Crowding ^4^	2.95	1.73	3.41	1.85	<0.001
Number of Children	3.95	1.86	4.52	1.95	<0.001
Number of Adults	2.49	1.09	2.33	0.87	0.007
Asset index ^5^ (log)	0.96	0.39	0.58	0.33	<0.001
**Community**					
Marginalization index ^6^	0.03	0.53	0.54	0.47	<0.001

Note: Data are mean and SD or percent, and are stratified by inclusion in the final sample. *p*-Values are generated from *t*-tests (for continuous variables) and chi-squared tests (for dichotomous variables) and adjusted for state of residence and clustering at the community level. ^1^ Indigenous community is defined as one in which more than 70% of the community population speaks an indigenous language. ^2^ Child development was measured using the Extended Ages and Stages Questionnaire (EASQ) and the Ages and Stages Questionnaire: Socio-emotional (ASQ:SE). ^3^ Education denotes the highest level completed. ^4^ Crowding is the number of people in the household divided by the number of rooms (including kitchen but not bathroom). ^5^ Asset index is a log of a PCA of a standard summary index of household possessions. ^6^ Community marginalization index is a composite of community economic indicators. EASQ: Extended Ages and Stages Questionnaire. ASQ: Ages and Stages Questionnaire.

**Table 2:** HOME ^1^ Inventory scores (*n* = 1893).

Scale	Mean	SD	Max Possible Score
Responsivity	7.52	2.69	11
Acceptance	5.81	1.52	8
Organization	4.06	1.25	6
Learning	2.46	2.05	9
Involvement	2.89	1.46	6
Variety	2.59	0.98	5
Total Score	25.32	6.08	45

Note: ^1^ HOME is the Home Observation for the Measurement of the Environment Inventory.

**Table 3:** Unadjusted and adjusted differences in HOME ^1^ Inventory scores between indigenous and non-indigenous communities (*n* = 1893).

Home Inventory Scale	Unadjusted					Adjusted ^2^				
	Non-Indigenous (*n* = 840)		Indigenous ^3^ (*n* = 1053)		Difference	Non-Indigenous (*n* = 840)		Indigenous ^3^ (*n* = 1053)		Difference
	Mean	SE	Mean	SE	*p*-Value	Mean	SE	Mean	SE	*p*-Value
Responsivity	8.30	0.13	6.89	0.09	<0.001	7.95	0.23	7.14	0.17	0.029
Acceptance	5.77	0.11	5.84	0.07	0.555	5.43	0.17	6.15	0.13	0.012
Organization	4.47	0.06	3.73	0.06	<0.001	4.32	0.08	3.84	0.08	0.001
Learning	3.13	0.10	1.92	0.10	<0.001	2.83	0.11	2.10	0.11	<0.001
Involvement	3.41	0.09	2.47	0.05	<0.001	3.31	0.11	2.53	0.07	<0.001
Variety	2.65	0.04	2.54	0.04	0.087	2.72	0.06	2.49	0.05	0.020
Total Score	27.73	0.33	23.40	0.23	<0.001	26.56	0.47	24.34	0.35	0.003

Note: ^1^ HOME is the Home Observation for the Measurement of the Environment Inventory. ^2^ Adjusted for child age and sex, parent education, father’s presence, household assets and crowding, family self-identification as indigenous, and community marginalization. ^3^ Indigenous community is defined as a community in which more than 70% of the population speaks an indigenous language. SE is the standard error of the population mean. *p*-Values correct for clustering at the state and community level.

**Table 4:** Stratified Ordinary Least Squares (OLS) analysis of characteristics associated with variability in total HOME ^1^ Inventory score: Indigenous ^2^ communities (*n* = 1053).

Variables	Unadjusted			Adjusted		
	β	SE	*p*-Value	β	SE	*p*-Value
Indigenous self-identification ^3^	1.70	0.91	0.065	1.59	0.89	0.078
Child age (months)	0.29	0.04	<0.001	0.28	0.04	<0.001
Child sex (girl)	0.33	0.35	0.345	0.12	0.31	0.697
Mother education ^4^						
Kindergarten or less	reference					
Primary	1.71	0.33	<0.001	1.46	0.36	<0.001
Secondary and above	3.18	0.65	<0.001	2.73	0.68	<0.001
Father education ^4^						
Kindergarten or less	reference					
Primary	1.09	0.43	0.012	0.32	0.40	0.417
Secondary and above	2.19	0.61	0.001	0.88	0.57	0.127
Father resides in home	0.11	0.59	0.859	0.52	0.55	0.347
Asset index ^5^						
<25%	reference					
25 to 51%	0.97	0.44	0.030	0.90	0.43	0.039
50 to 74%	2.26	0.49	<0.001	1.99	0.48	<0.001
≥75%	1.84	0.67	0.007	1.64	0.65	0.014
Household crowding ^6^	−0.31	0.10	0.003	−0.11	0.11	0.321
Community marginalization index ^7^	−0.29	0.50	0.567	0.56	0.50	0.354
R^2^				0.099		

Note: ^1^ HOME is the Home Observation for the Measurement of the Environment Inventory. ^2^ Indigenous community is defined as a community in which more than 70% of the population speaks an indigenous language. ^3^ Family self-identification as indigenous. ^4^ Education denotes the highest level completed. ^5^ Asset index is a log of a PCA of a standard summary index of household possessions. ^6^ Household crowding is the number of people in the household divided by the number of rooms (including kitchen but not bathroom). ^7^ Community marginalization index is a composite of community economic indicators. SE is the standard error of the estimate. *p*-Values correct for clustering at the state and community level.

**Table 5:** Stratified OLS analysis of characteristics associated with variability in total HOME ^1^ Inventory score: Non-indigenous ^2^ communities (*n* = 840).

Variables	Unadjusted			Adjusted		
	β	SE	*p*-Value	β	SE	*p*-Value
Indigenous self-identification ^3^	−1.87	0.85	0.029	−092	0.74	0.218
Child age (months)	0.22	0.05	<0.001	0.21	0.04	<0.001
Child sex (girl)	−0.48	0.37	0.190	−0.45	0.35	0.208
Mother education ^4^						
Kindergarten or less	reference					
Primary	0.21	0.57	0.709	0.06	0.56	0.912
Secondary and above	2.24	0.67	0.001	0.87	0.70	0.215
Father education ^4^						
Kindergarten or less	reference					
Primary	−0.89	0.65	0.171	−1.46	0.58	0.013
Secondary and above	0.51	0.73	0.487	−1.00	0.68	0.145
Father resides in home	0.62	0.64	0.331	0.88	0.55	0.115
Asset index ^5^						
<25%	reference					
25 to 49%	1.66	1.02	0.105	1.73	0.92	0.061
50 to 74%	3.60	1.12	0.002	3.04	0.96	0.002
≥75%	5.30	1.11	<0.001	4.14	0.93	<0.001
Household crowding ^6^	−0.62	0.12	<0.001	−0.38	0.12	0.002
Community marginalization index ^7^	−2.56	0.67	<0.001	−0.96	0.63	0.132
EASQ Global score ^8^	2.25	0.22	<0.001			
ASQ Socio-emotional score ^8^	1.60	0.25	<0.001			
R^2^				0.192		

Note: ^1^ HOME is the Home Observation for the Measurement of the Environment Inventory. ^2^ Indigenous community is defined as a community in which more than 70% of the population speaks an indigenous language. ^3^ Family self-identification as indigenous. ^4^ Education denotes the highest level completed. ^5^ Asset index is a log of a PCA of a standard summary index of household possessions. ^6^ Crowding is the number of people in the household divided by the number of rooms (including kitchen but not bathroom). ^7^ Community marginalization index is a composite of community economic indicators. ^.8^ Child development was measured using the Extended Ages and Stages Questionnaire (EASQ) and the Ages and Stages Questionnaire: Socio-emotional (ASQ:SE).

**Table 6:** Effect modifiers of the OLS model relationship between indigenous community ^1^ and total HOME ^2^ Inventory score (*n* = 1893).

Variables	Model 1			Model 2			Model 3			Model 4		
	β	SE	*p*	β	SE	*p*	β	SE	*p*	β	SE	*p*
Indigenous community (IC)	−4.25	0.98	<0.001	−3.95	0.62	<0.001	−4.36	0.68	<0.001	−1.31	1.04	0.207
Indigenous self-identification ^3^	−1.58	0.83	0.06									
IC × indigenous self-identification	2.96	1.25	0.019									
Mother’s education ^4^												
Kindergarten or less				reference								
Primary				−0.1	0.55	0.861						
Secondary and above				1.12	0.66	0.093						
IC × primary				1.54	0.65	0.018						
IC × secondary				1.45	0.89	0.102						
Father’s education ^4^												
Kindergarten or less							reference					
Primary							−1.65	0.61	0.007			
Secondary and above							−0.95	0.68	0.165			
IC × primary							1.99	0.72	0.006			
IC × secondary							1.67	0.88	0.058			
Asset index ^5^												
<25%										reference		
25 to 49%										1.72	0.98	0.08
50 to 74%										3.3	1.05	0.002
≥75%										4.6	1	<0.001
IC × 25 to 49%										−0.87	1.07	0.416
IC × 50 to 74%										−1.36	1.14	0.235
IC × ≥75%										−3.14	1.17	0.008

Note: Modifiers of the association between living in an indigenous community and family self-identification as indigenous, mother and father’s education, and household assets were run as separate models. The models were adjusted for demographic characteristics (child age and sex, mother and father education, father’s presence, household assets and crowding, family self-identification as indigenous, and community marginalization). ^1^ Indigenous community is defined as one in which more than 70% of the population speaks an indigenous language. ^2^ HOME is the Home Observation for the Measurement of the Environment Inventory. ^3^ Family self-identification as indigenous. ^4^ Education denotes the highest level completed. ^5^ Asset index is a log of a PCA of a standard summary index of household possessions. *p*-Values are adjusted for state of residence and clustering at the community level.
